# Curiosity as compass: a conversation with David Attwell

**DOI:** 10.1117/1.NPh.13.2.020401

**Published:** 2026-05-14

**Authors:** Anusha Mishra

**Affiliations:** Oregon Health & Science University, Jungers Center for Neurosciences Research, Department of Neurology, Portland, Oregon, United States

## Abstract

Anusha Mishra interviewed David Attwell, Jodrell Professor of Physiology at University College London, whose work has been foundational to our understanding of glial physiology, neurovascular coupling, and vascular contributions to dementia. A video of the interview is available: https://doi.org/10.1117/1.NPh.13.2.020401.

**Figure:**
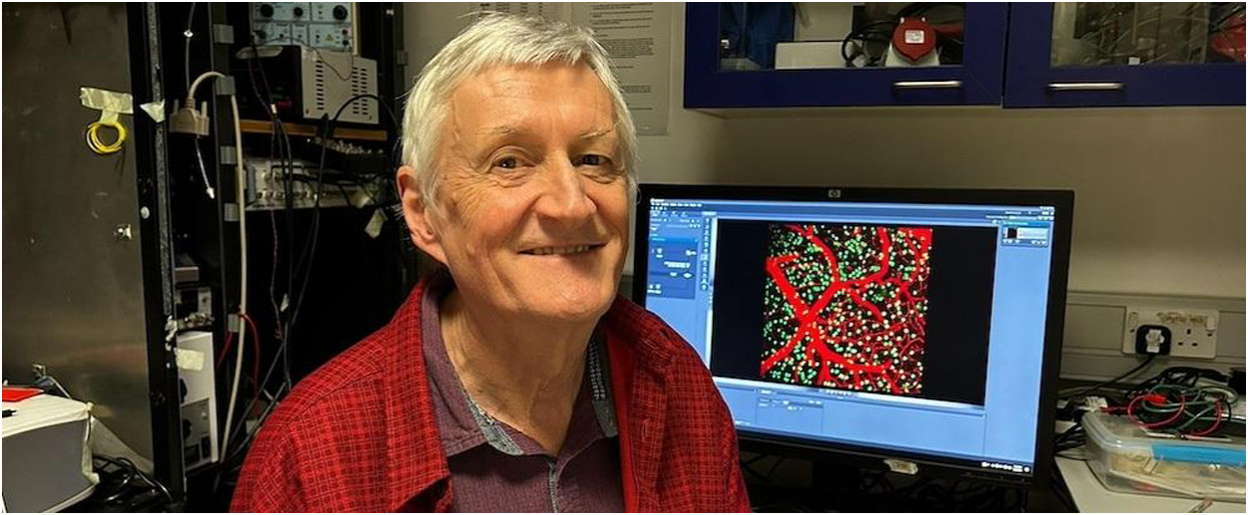
David Attwell is the Jodrell Professor of Physiology at University College London. His work has been foundational to our understanding of glial physiology, neurovascular coupling, and vascular contributions to dementia. View a video of our conversation at https://doi.org/10.1117/1.NPh.13.2.020401.

Scientific careers rarely unfold according to a strict plan. More often, they are shaped by curiosity, unexpected results, and a willingness to follow questions wherever they lead. Few neuroscientists exemplify this better than David Attwell. Over several decades, his work has helped redefine how we think about glial cells, cerebral blood flow, and the vascular underpinnings of neurological disease.

In a recent conversation with David—who also happens to be my former postdoctoral mentor—we discussed the winding path that took him from physics to neuroscience, the experiments that changed the direction of his lab, and the principles that have guided his approach to science and mentorship.

## From Physics to Physiology

David did not begin his academic life in biology. As an undergraduate at Oxford, he studied physics and thoroughly enjoyed it. Yet when he spoke with people pursuing PhDs in the field, the mood was bleak. Physics, he was told, was “dead.” In contrast, his friends studying biology and medicine were enthusiastic and visibly happy. That difference planted the first seed of doubt about his future direction.

Another realization followed: a background in mathematics and physics could provide a real advantage in biology. David found a mentor willing to take a chance on him—Julian Jack, then a leading figure in modeling neuronal electrical signaling—even though David had virtually no biology training. Reading John Eccles’ *The Cerebellum as a Neuronal Machine* further convinced him that biological systems were fascinating.

With financial support from his college, David completed an intensive one-year physiology degree and transitioned fully into neuroscience. He worked in both Julian Jack’s lab and Denis Noble’s cardiac electrophysiology group, experiences that grounded him deeply in experimental physiology and electrophysiology.

## Retinas, Transporters, and Being Proven Wrong

After his PhD, David moved to the University of California, Berkeley, to work with Frank Werblin on retinal circuitry and visual information processing. When he later established his own lab at University College London, he continued working on the retina—until an experiment he expected to fail changed everything.

One of his PhD students suggested investigating glutamate uptake in Müller glial cells. Based on simple calculations and prevailing assumptions, David was skeptical; the expected signals seemed impossibly small. The student proceeded anyway—and recorded currents hundreds of times larger than anticipated. That single observation redirected the lab for years, leading to foundational work on glutamate transport, excitotoxicity, and the active role of glia in brain function.

This pattern—hypotheses challenged by data—became a defining feature of the lab. Over time, projects expanded beyond neurons to astrocytes, oligodendrocytes, and microglia. This was not part of a master plan, David emphasized, but a consequence of how he runs a research group: recruit excellent people and give them the freedom to pursue questions they find genuinely interesting.

## Tools in Service of Questions

As the science evolved, so did the tools. David’s lab adopted patch-clamp electrophysiology early, benefitting from local connections when the technique was first developed. Two-photon imaging followed soon after. Rather than emphasizing technological novelty for its own sake, the goal was always to use new methods when they enabled better questions to be asked.

Whenever possible, David favored approaches that allowed the lab to focus on experiments rather than engineering. The priority was progress and insight, not building systems from scratch unless necessary.

## Pericytes and the Microvasculature

One of the most consequential shifts in the lab’s research direction emerged almost by accident. While patch clamping cells in the retina, a student frequently encountered cells associated with blood vessels rather than neurons. These turned out to include pericytes—cells that had been described in the nineteenth century as contractile but whose functional role had largely been forgotten.

Simple experiments demonstrated that depolarizing pericytes could strongly constrict capillaries. This suggested that blood flow regulation occurs not only in arteries and arterioles but deep within capillary networks themselves. The implications were profound, particularly for disease.

I recall being deeply skeptical when I first encountered this work. Rather than being offended, David encouraged me to test the idea rigorously. I did—and, like many others before me, I was convinced by the evidence. That openness to being challenged, provided the challenge is experimental, remains one of the most influential aspects of his mentorship.

## Mentorship, Credit, and Lab Culture

Long before it became common practice, David made a point of explicitly crediting students and postdocs during talks, often naming them and showing their photographs alongside the data they generated. His rationale was straightforward: they did the work, and they deserved to be seen.

Over 25 years directing the neuroscience PhD program at UCL, David guided more than a hundred and fifty trainees into research careers. He emphasized that mentorship must be individualized—some people need close guidance, others independence—and that personal challenges are inevitable. When conflicts arise, he believes in addressing them directly and humanely, before resentment takes hold.

David spoke candidly about how academia has changed. Administrative burden has grown, and the simple joy of discovery can be harder to protect. His advice to early-career scientists is pragmatic: write fewer grants but make them better, and build a trusted network of colleagues who will give honest, sometimes uncomfortable feedback.

## Blood Flow and Dementia

In recent years, David has focused much of his attention on vascular contributions to dementia. Reduced cerebral blood flow, he explained, is not a secondary feature but a central component of both Alzheimer’s disease and vascular dementia. In Alzheimer’s disease, affected brain regions can experience reductions in blood flow approaching 50%, with major consequences for neuronal function and protein synthesis.

Encouragingly, some interventions that restore blood flow do more than slow decline; they improve cognition. This has major implications for how we think about treatment strategies, suggesting that vascular health is not merely supportive, but therapeutic.

## Advice for the Next Generation

When asked what advice he would offer young scientists facing uncertainty, imposter syndrome, and external pressures beyond their control, David’s response was simple: do what excites you most. Funding realities matter, but curiosity is what sustains a scientific career over decades.

David Attwell’s career is a reminder that the most influential science often arises not from rigid plans, but from paying close attention—to data, to people, and to questions that refuse to be ignored.

